# Add fuel to the fire: Inflammation and immune response in lung cancer combined with COVID-19

**DOI:** 10.3389/fimmu.2023.1174184

**Published:** 2023-03-23

**Authors:** Yanling Ai, Hengyi Wang, Qiao Zheng, Songtao Li, Jingwen Liu, Ju Huang, Jianyuan Tang, Xiangrui Meng

**Affiliations:** ^1^ Department of Oncology, Hospital of Chengdu University of Traditional Chinese Medicine, Chengdu, China; ^2^ Department of Infectious Diseases, Hospital of Chengdu University of Traditional Chinese Medicine, Chengdu, China; ^3^ Traditional Chinese Medicine (TCM) Regulating Metabolic Diseases Key Laboratory of Sichuan Province, Hospital of Chengdu University of Traditional Chinese Medicine, Chengdu, China; ^4^ Clinical School of Medicine, Chengdu University of Traditional Chinese Medicine, Chengdu, China

**Keywords:** lung cancer, COVID-19, inflammation, immunity, immune checkpoint inhibitor

## Abstract

The corona virus disease 2019 (COVID-19) global pandemic has had an unprecedented and persistent impact on oncological practice, especially for patients with lung cancer, who are more vulnerable to the virus than the normal population. Indeed, the onset, progression, and prognosis of the two diseases may in some cases influence each other, and inflammation is an important link between them. The original chronic inflammatory environment of lung cancer patients may increase the risk of infection with COVID-19 and exacerbate secondary damage. Meanwhile, the acute inflammation caused by COVID-19 may induce tumour progression or cause immune activation. In this article, from the perspective of the immune microenvironment, the pathophysiological changes in the lungs and whole body of these special patients will be summarised and analysed to explore the possible immunological storm, immunosuppression, and immune escape phenomenon caused by chronic inflammation complicated by acute inflammation. The effects of COVID-19 on immune cells, inflammatory factors, chemokines, and related target proteins in the immune microenvironment of tumours are also discussed, as well as the potential role of the COVID-19 vaccine and immune checkpoint inhibitors in this setting. Finally, we provide recommendations for the treatment of lung cancer combined with COVID-19 in this special group.

## 1 Introduction

Lung cancer is the second most diagnosed cancer and the leading cause of cancer deaths worldwide, to which 1.8 million deaths were attributed in 2020 (1, 2). As a public health concern, chronic inflammation is an important mechanism of lung cancer initiation, progression, and metastasis. Meanwhile, in patients with lung cancer, the long-term secretion of inflammatory substances also creates a special tumour immune microenvironment (TIME) and lung pathological changes (3). Cytotoxic chemotherapy has long been the main route of systemic drug therapy for lung cancer; however, it also destroys the body’s immune defence system while killing cancer cells. With the development of therapeutic technologies, targeted therapy and immunotherapy allow more patients to gradually achieve long-term tumorigenic survival through site-directed attack and immune activation.

In late 2019, there was an outbreak of corona virus disease 2019 (COVID-19) and a rapid worldwide mass spread ensued. As a single positive-strand RNA virus, severe acute respiratory syndrome coronavirus 2 (SARS-CoV-2), the causative agent of COVID-19, is constantly subject to variation, and currently, variants such as Alpha, Beta, Gamma, Delta, and Omicron are mainly found (4). The infection and replication of the virus provoke an acute inflammatory response, which can promote body self-protection and damage repair. However, an imbalanced immune response may also destroy normal tissues, causing acute lung injury, multiple organ failure, and even death. Among the various subgroups of patients, tumour patients are a special population who are often under systemic immunosuppression due to the primary lesion and anticancer treatment (5). Lung cancer patients are more susceptible to COVID-19 pneumonia than the normal population (6).

**Figure 1:**
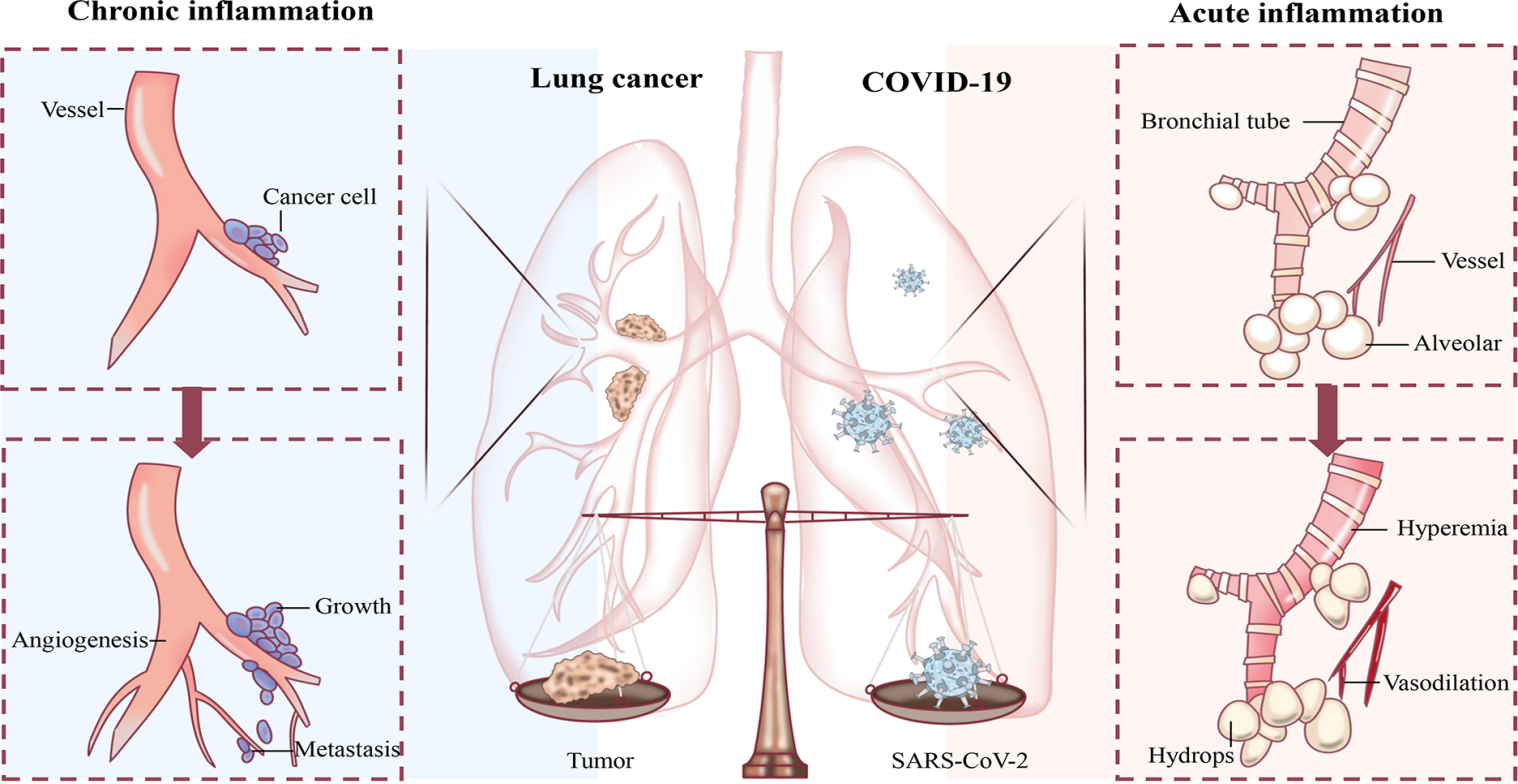
Schematic diagram of the influence of inflammation on lung cancer and COVID-19. Chronic inflammation creates a suitable environment for lung cancer growth, proliferation, metastasis, as well as angiogenesis. The acute inflammation inflicted by COVID-19 dilates the alveolar wall and interstitial blood vessels with increased permeability, causing the accumulation of serous fluid in the alveolar space.

Indeed, the tumour cells themselves are in the TIME, and in this unhealed wound, a moment is accompanied by a chronic inflammatory response (7). Therefore, with the introduction of COVID-19, in addition to the original chronic inflammatory basis, patients with lung cancer were also faced with SARS-CoV-2-mediated activation of many immune cells, which led to host-pathogen interactions different from those in normal individuals (**Figure 1**). This article describes the changes and potential mechanisms of TIME after the consolidation of COVID-19 in lung cancer patients and discusses the therapeutic protective measures for this special population, hoping to provide a reference for future studies.

## 2 Lung cancer and chronic inflammation

**Figure 2:**
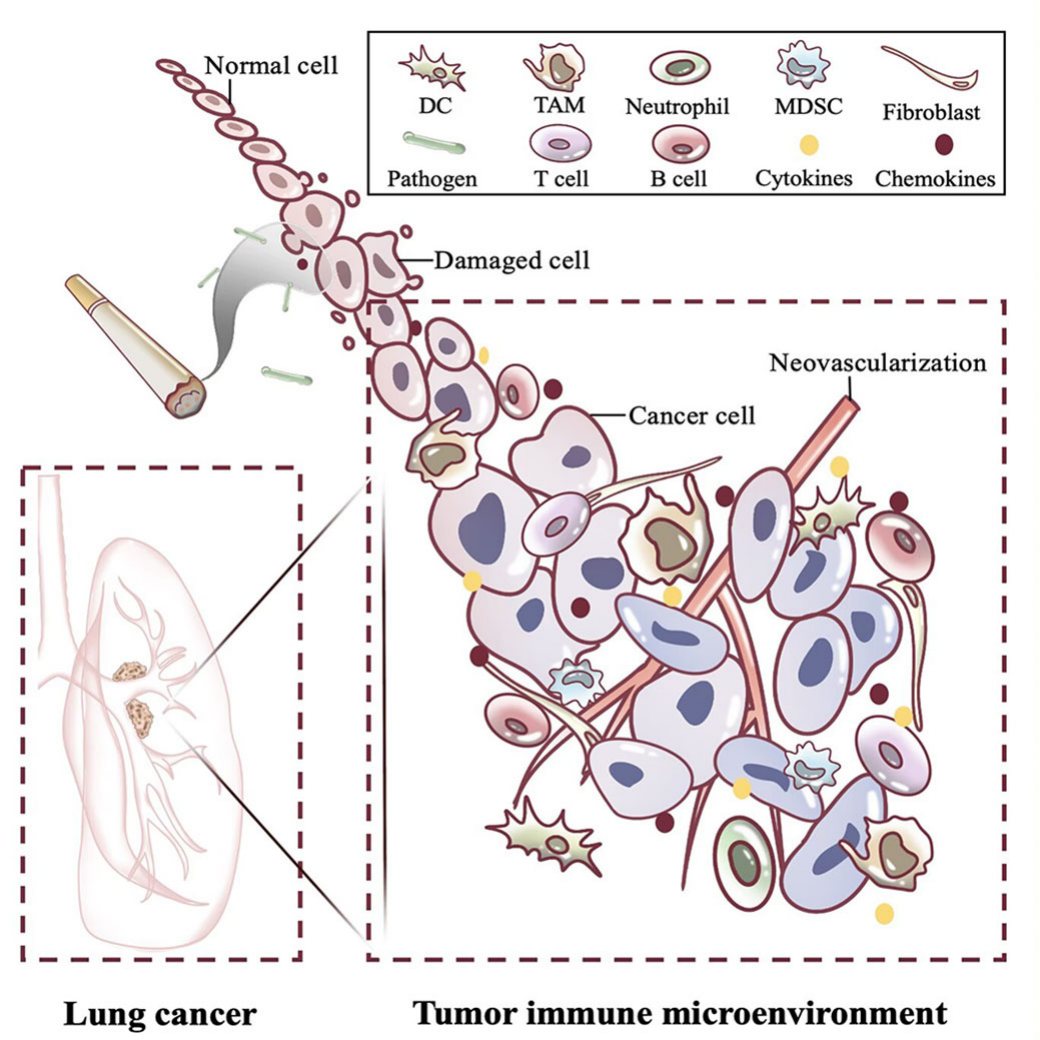
Changes in immune microenvironment of lung cancer caused by chronic inflammation. The long-term presence of carcinogenic factors such as smoking and pathogens causes lung cancer and shapes the tumor immune microenvironment. Around the tumor, inflammatory cells massively infiltrate and recruit cytokines and chemokines, which promote immune escape of the tumor through complex interactions. DC, Dendritic cell; TAM, Tumor macrophage; MDSC, Myeloid-derived suppressor cell; T cell, T lymphocyte cell; B cell, B lymphocyte cell.

The molecular pathways involved in lung cancer remain poorly defined, but in general, smoking, occupational and environmental exposure, air pollution, ionizing radiation are mainly implicated. The above external factors can induce gene alterations and cell malignant transformation, such as activating oncogenes KRAS, EGFR, ALK1 (8, 9) and silencing tumor suppressor genes P53, Rb1, UTX (10, 11). In addition, The lungs communicate with the external environment. To this end, the respiratory system forms a sophisticated set of defence mechanisms to filter or clear various adverse factors, such as secretory fluid and cilia within the lining of the respiratory tract, cough reflex, and alveolar surface proteins (12). However, the continuous occurrence of multiple types of infection sources in nature makes damage to lung tissue difficult to avoid, such as pathogenic microorganisms, toxins, pollutants, irritants, and allergenic sources. This enables a complex immune response between pro- and anti-inflammatory factors and contributes to tissue repair and organ damage as well as secondary pathophysiological alterations (**Figure 2**).

### 2.1 Occurrence of lung cancer

In the 1990s, Rudolf Virchow proposed the hypothesis that chronic inflammation may induce tumours based on observations from tumour biopsies (13). Studies have confirmed that the occurrence of lung cancer is closely related to chronic inflammatory diseases of the lungs, and there is an association between the use of nonsteroidal anti-inflammatory drugs and a reduction in the incidence of lung cancer (14). Lung diseases, such as chronic obstructive pulmonary disease, tuberculosis, and interstitial lung disease, increase the risk of lung cancer. Long-term exposure to various pollutants such as external pathogens, tobacco smoke, and asbestos fibres may cause the release of various cytokines and growth factors that provide a selective growth advantage to mutant cells (15–17). In lung cancer, due to endogenous and exogenous high-risk factors, chronic inflammation is a common and important pathogenesis. Chronic inflammation in the lung can induce the accumulation of inflammatory cells, which produce many inflammatory cytokines and chemotactic molecules such as interleukin (IL)-1, IL-2, IL-6, IL-8, tumour necrosis factor-α (TNF-α), and cyclooxygenase 2 (COX2) (3). Their persistence and the cascade they cause can induce normal cell damage and immune dysregulation and create an intracellular environment that favours genotoxic damage and lung carcinogenesis (18, 19).

### 2.2 Progression of lung cancer

Chronic inflammation triggers lung carcinogenesis and is an important driver of lung cancer progression. Faget et al. (20) found that in lung cancer models, neutrophils, tumour-associated macrophages, and T and B cells infiltrated greatly outside the tumour tissue, which was beneficial to tumour growth. Inflammatory cells together with fibroblasts, endothelial cells, and extracellular matrix in the tumour stroma constitute TIME. TIME has a pro-tumorigenic effect. Developing tumour cells produce cytokines and chemokines to recruit inflammatory components, such as leukocytes (21). In addition, accumulated genetic changes may contribute to the malignant development of some tumours, and the chronic inflammatory response is considered an important endogenous source of mutational events (22). The inflammatory component shapes the potentially genotoxic environment by releasing chemicals, especially the sustained expression of COX-2, to maintain the abnormal status of tumours and accelerate their malignant evolution (23).

### 2.3 Metastasis of lung cancer

The delicate balance between inflammation and anti-inflammation is essential for the homeostatic maintenance of the lungs. In tumour patients, the TIME boundary is chronically populated with inflammatory cells, which produce extracellular matrix degrading enzymes such as matrix metalloproteinases and other pro-invasive growth factors (24, 25). For example, macrophages can suppress antitumor immunity, stimulate angiogenesis, and tumour escape, while preparing the target tissue for the arrival of tumour cells at the site of invasion (25). Lung cancer activates macrophages *via* Toll-like receptor (TLR) family member TLRs and produces TNF-α, and the creation of an inflammatory environment favours the occurrence of metastasis (26). In lung cancer tissue, the chemokine receptor system is also significantly altered, and in addition to regulating the inflammatory state, exerts direct effects on tumour progression, as CXCR2 ligands are closely associated with vascular growth and spontaneous migration (27). At later stages of the tumorigenic process, in the face of high levels of chemokines and cytokines, the host immune response is weakened or blunted, and failure to upregulate anti-inflammatory cytokines allows tumour cells to evade immune destruction and achieve infiltration and metastasis.

In recent years, studies on the relationship between inflammation and lung cancer have been in-depth. We consider the need to further identify the most critical inflammatory drivers affecting the TIME, taking into account the inflammatory response in different patients, in order to more precisely improve the efficacy of lung cancer treatment.

## 3 Three phases of COVID-19 and acute inflammation

**Figure 3:**
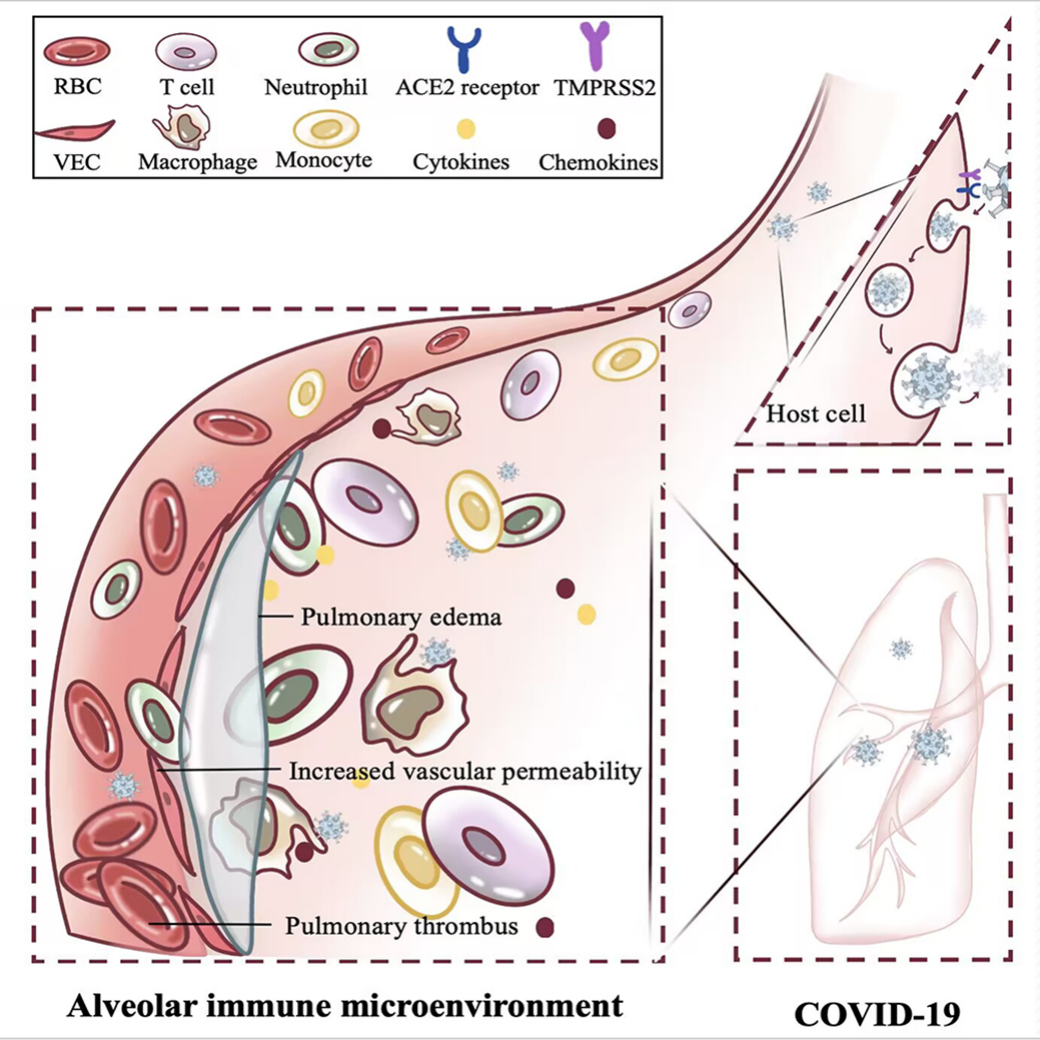
Effect of acute inflammation on alveolar immune microenvironment. The rapid replication of SARS-CoV-2 within the host induces acute inflammation in the lung, confers increased vascular permeability, heightened thrombotic risk, and triggers pulmonary edema. RBC, Red blood cell; T cell, T lymphocyte cell; ACE2, Angiotensin-converting enzyme 2; TMPRSS2, Transmembrane serine protease 2; VEC, Vascular endothelial cell.

COVID-19 is caused by SARS-CoV-2 and is mainly divided into three temporal phases: acute infection, post-acute hyperinflammatory disease, and late inflammatory and viral sequelae (28). For most young and healthy people, the adverse effects caused by COVID-19 are relatively mild and have a good prognosis; however, a few patients have rapid progression and are critically ill, mostly seen in older individuals with chronic underlying diseases (29) (**Figure 3**).

### 3.1 Acute infection

The duration of acute infection typically ranges from days to weeks (30). At disease onset, viral replication in the body triggers an initial host immune response with positive real-time reverse transcription quantitative polymerase chain reaction (RT-qPCR) and rapid antigen testing. Patients present with typical flu-like symptoms such as fever, cough, and shortness of breath (31). In mild-to-moderate SARS-CoV-2-infected individuals, IL-6, IL-10, and TNF-α levels were mildly increased or within the normal range during the acute inflammatory phase and decreased during recovery (32). Typically, type I interferon (IFN)-α/β is the first line of defence that restricts virus replicative propagation upon entering the body and is mainly derived from uninfected cells such as resident macrophages and other phagocytic cells (33). However, human coronavirus (hCoV) can inhibit the IFN-α/β-mediated innate immune response (34, 35), making it either a delayed response or severely impaired (36). Inside the organism, an inadequate early front-line defence mechanism gives a viral multiplier, promotes COVID-19 progression, and induces an acute inflammatory response that can also lead to macrophage activation syndrome-like pathology, posing a hidden risk for subsequent acute post-hyperinflammatory diseases (37).

### 3.2 Post-acute hyperinflammatory disease

Following acute infection, a minority of patients develop systemic inflammation, and although RT-qPCR testing may be negative in patients at this time, the virus continues to replicate *in vivo* (29). Among them, in severe COVID-19 patients requiring intensive care unit (ICU) admission, the condition may be further exacerbated by massive inflammatory cell infiltration and high levels of proinflammatory cytokines and chemokines (38, 39), which in severe cases can progress to acute lung injury, acute respiratory distress syndrome (ARDS), or multiple organ failure (40). Respiratory failure due to ARDS is the leading cause of patient mortality (41). The lungs of these patients harbour a highly proinflammatory macrophage microenvironment (42), exhibiting significant elevation of IL-6, IL-8, IL-10, and TNF-α, and a decrease in CD4+ and CD8+ T cells, triggering a cytokine storm (32, 43, 44). In addition to a significant decrease in T cell counts, patients showed increased expression of PD-1 and Tim-3 on T cells, indicating that surviving T cells were functionally exhausted (45). This suggests that the cytokine storm may have suppressed the body’s adaptive immunity to viral infection.

### 3.3 Late inflammation and virological sequelae

Later in COVID-19 infection, the viral load decreases in the patient, and the associated symptoms generally resolve. However, increasing evidence suggests that in some patients after SARS-COV-2 infection, malaise, including tachycardia, fatigue, pain, and brain fog (46, 47). The aetiology of such symptoms is not fully defined but may be associated with the persistence of the later inflammatory state. Studies have found that peak viral levels remain detectable in plasma samples up to 12 months after diagnosis in patients with confirmed COVID-19 (48). Despite the ability of SARS-COV-2 to induce a systemic antiviral response, it also causes persistent inflammatory pathology well beyond the clearance of the primary infection (47). In addition, proinflammatory cytokines may establish a pathological proinflammatory feedback loop (TLR4/rage loop) during acute infection that remains operative even after viral clearance (49). This allows multiorgan and multisystem involvement and affects patient emotional status. Furthermore, Gold et al. (50) suggests that the prolonged presence of sequelae may not be a direct effect of SARS-COV-2 infection but a consequence of inflammation-induced Epstein–Barr virus reactivation in COVID-19.

Given the important role of inflammation in SARS-COV-2 infection, we speculate that combining antiviral and anti-inflammatory drugs may offer advantages over single agents, and natural products may provide inspiration in this regard. We believe that a comprehensive understanding of the immunological mechanism of infection and combining it with diagnosis and treatment will greatly advance the prevention and treatment of COVID-19.

## 4 Lung cancer patients with COVID-19

As of January 31, 2023 the World Health Organization has accumulated notifications of 753,479,439 confirmed cases and 6,812,798 deaths worldwide from COVID-19 (51). Owing to the limitations of detection capabilities across regions and the impact of death attribution, the true infection and mortality rates are likely to far exceed official statistics. In lung cancer patients, the presence of one or more additional comorbidities, such as age at presentation > 65 years, history of smoking, and presence of chronic obstructive pulmonary disease, can be confounded by the presence of several risk factors (52). This has led to increased morbidity and mortality when confronted with COVID-19.

### 4.1 Potential immunological impact of chronic inflammation in lung cancer on COVID-19

#### 4.1.1 Increased risk of infection

Generally, inflammation is an organism’s resistance response to disease that is somewhat self-limiting, depending on the presence or absence of the threat. However, due to the presence of certain social, psychological, environmental, and biological factors, inflammation fails to resolve normally and instead converts into a low-grade, non-infectious chronic state (53). This distinguishes the immune components involved in the response from acute inflammation (54). The presence of chronic inflammation can lead to a breakdown of immune tolerance, impair the normal physiological function of tissue organs, and make the body more susceptible to infection, as well as a low response to vaccines (55). Patients with other underlying lung diseases, such as lung cancer, are more susceptible to infection because of the reduced clearance of the virus. Additionally, angiotensin-converting enzyme 2 (ACE2) and transmembrane serine protease 2 (TMPRSS2) are key molecules involved in the spread of SARS-CoV-2 (56). Viruses use the S protein to bind ACE2 on the surface of human cells to enter host cells, and TMPRSS2 promotes S protein activation, triggering fusion of viral and cellular membranes (56). Expression of ACE2 and TMPRSS2 is elevated in lung cancer survivors compared to non-cancer individuals (57, 58). This may be related to the susceptibility of lung cancer patients to the SARS-CoV-2 virus. Interestingly, the examination of resected tissue specimens (containing both tumor and normal part) from non-small cell lung cancer (NSCLC) patients found that ACE2 transcript detected in tumor part did not correlate with disease stage, whereas expression in normal part was higher in individuals with advanced stages (59).This suggests that normal lung tissue (but not tumour tissue) in NSCLC patients may be key to distinguishing between the expected low and high risk of severe COVID-19.

#### 4.1.2 Exacerbated secondary injury

T cell antigen-mediated cellular immunity and humoral immunity can be provoked after SARS-CoV-2 infection in patients. An appropriate and timely immune response in most patients enables efficient clearance of SARS-CoV-2 and control of acute infection. Some groups experience prolonged exacerbation, which can be caused by excessive immune and inflammatory responses and is referred to as a cytokine storm. In lung cancer patients, chronic inflammation in the lung can cause a surge in proinflammatory immune responses, resulting in increased cytokine secretion by T cells and phagocytes (60). The TIME instead supports the SARS-CoV-2 protein by activating a cytokine storm and cell metabolism variant-related pathways, further accelerating the infection and weakening the immune system (61, 62). Compared to the general population, patients with lung cancer experience a further reduction in lung volume caused by the presence of neoplasias, which enables an earlier time to adverse effects in the case of inflammation (63). In addition, smoking, the most important high-risk factor for lung cancer (2), is associated with the severity of COVID-19 (64, 65). Smith et al. (66) found that chronic smoke exposure causes ACE2 content-dependent upregulation in the lungs of humans and animals, with protective expansion of mucus-secreting goblet cells. As a binding target of SARS-CoV-2, higher levels of ACE2 expression tend to be observed in the lung tissue of patients with severe COVID-19 (67). Downregulation of ACE2 due to binding may drive increased angiotensin II activity, contributing to pulmonary vasoconstriction and inflammatory and oxidative organ damage, thus contributing to acute lung injury and other systemic effect risks (68).

### 4.2 Potential immunological impact of acute inflammation of COVID-19 on lung cancer

#### 4.2.1 Induction of tumour progression

Viral infection is thought to promote the growth of human cancers (69). Yan et al. (70) found that acute lung infection can significantly affect cancer cell homing to the lungs and lung metastasis, which may be associated with an altered lung immune microenvironment. The potential effects of the long-term prevalence of COVID-19 on lung cancer patients remain unknown, but some scholars propose (71, 72) that during acute inflammation, the immune homeostasis of tissues is disrupted, and severe COVID-19 may seriously worsen the prognosis of lung cancer patients by accelerating tumour progression (73), possibly induce the reactivation of dormant cancer cells, and increase the risk of cancer recurrence. For lung cancer patients, ACE2 and the renin-angiotensin system to which it belongs can inhibit tumour cell growth, control inflammation, and VEGF production, thus maintaining vascular homeostasis (74). Reduction of ACE2 by virus binding may then lead to NF-κB overactivation, stimulate angiogenesis and immunosuppression over time, and promote neoplastic processes (74, 75). Moreover, in patients with severe COVID-19, the cytokine storm is mainly associated with a massive production of proinflammatory cytokines (i.e. IL-1β, IL-6, and TNF-α) (44, 76). IL-1β can create an inflammatory microenvironment that favours tumour initiation and promotion, leading to an increased risk of lung cancer. Similar to IL-1β, high levels of IL-6 may contribute to the growth, metastasis, and immune escape of lung cancer cells (77).

#### 4.2.2 Improved control condition

Severe COVID-19 is considered an acute hyperinflammatory disease characterised by massive immune cell activation, and its resulting lung infection has a certain negative impact on lung cancer patients. However, cases of an improved immune response to tumours after COVID-19 infection in sporadic cancer patients (78, 79), or even reports of improved responses to refractory cancer after mRNA vaccination, have also appeared (80). This may be related to the fact that, in the context of acute inflammation, rapid fluctuations in cytokines allow the immune system to be reactivated. For example, IFN-α can increase CD27+ and CD8+ T cell infiltration in the TIME to combat immune escape through competition for glucose metabolism (81). Additionally, it was found that the S1 protein of SARS-CoV-2 can activate the NF-κB signalling pathway in lung cancer cells, increase the expression of proinflammatory factors such as TNF-α, and thus induce lung cancer cell death (82). Second, COVID-19 acute infection prompts neutrophils to be recruited to the lungs in prophase, which may be a source of excessive neutrophil extracellular traps (NETs) (83). In mouse models inoculated with Lewis lung carcinoma, large areas of necrotic neutrophils and NET-like structures would release cytotoxic substances, impairing tumour vascular integrity (84). Additionally, during vaccination, CD4+ T cells and exhausted CD8+ T cells may also be reactivated and in turn prevent myeloid-derived suppressor cells or recruit regulatory T cells, exerting antitumor effects (80). Notably, there are many common signalling pathways between COVID-19 and lung cancer (85), and it is difficult for individual case reports to comprehensively reflect the correlation between various complex effects. So we still recommend cancer patients to be protected and avoid active or passive infection.

### 4.3 Potential immunological impact of COVID-19 vaccine in lung cancer

The COVID-19 vaccine is an effective way to prevent SARS-CoV-2 infection, stimulating the formation of virus-neutralising antibodies while inducing cellular immunity (86). With the marketing and popularity of different types of vaccines (87), the Society of Oncology Science recommends preferential vaccination of patients with cancer (88, 89). However, there is limited information on the safety and efficacy of the vaccine in patients with cancer because vulnerable populations, including immunocompromised patients, are not involved in research development and clinical trials of the vaccine (90). Previous evidence (91) shows that among those using immunosuppressive drugs, less than half of the vaccines developed sufficient levels of antiviral antibodies, and that the mean antibody level in positive patients was two-fold lower than that in healthy controls. As a key population in which normal immunity is compromised, early studies have argued that cancer patients have impaired neutralising IgG responses to mRNA vaccines and reduced neutralising antibody responses (92). In addition, a study found that during anti-CTLA4 and anti-PD-1 combination immunotherapy, a lung cancer patient developed cytokine release syndrome due to a new crown vaccination, elevated cytokine levels (IL-6, IL-10, and IFN-γ), and grade 2 liver and kidney dysfunction (93). However, Hernandez et al. (94) believe that the COVID-19 vaccine is safe for patients with lung cancer, and most patients can generate immunity after the first and second doses, reducing the risk of infection.

Harada et al. (95) also provided safety data on the use of the COVID-19 vaccine in patients with advanced lung cancer receiving anticancer treatments, such as chemotherapy, immunotherapy, and targeted therapy, while also noting that vaccine-related side effects tend to increase in patients with lung cancer receiving cytotoxic chemotherapy. Therefore, we call for more adequate studies focusing on the effects of vaccines on humoral and cellular immunity in lung cancer patients.

## 5 Treatment of lung cancer combined with COVID-19

Currently, the COVID-19 pandemic promotes the development of health care systems and severely affects the care of patients with cancer. In lung cancer patients receiving antineoplastic therapy, COVID-19 may interfere with their original treatment plan. For example, chemotherapy may weaken the anti-disease ability of the immune system for a short time, and molecular targeted drugs or immunotherapy may trigger inflammatory changes in the lungs (96). In response to this, it is recommended that the risk-benefit ratio of the various treatments of COVID-19 should be thoroughly discussed with patients and arranged based on clinical prioritisation. For example, given the risk of infection posed by surgical resection and the potentially immunosuppressive state triggered by perioperative chemotherapy, reassessment of therapy is warranted for individual patients.

Several institutions have conducted studies on the impact of various anticancer treatments received by lung cancer patients combined with COVID-19, but these are limited by sample size, geographic region, and diagnostic discrimination and data timeliness; the results obtained from preliminary reports are not yet in agreement. Calles et al. (97) found that COVID-19 morbidity and mortality were higher among lung cancer patients receiving immune checkpoint inhibitors, but no statistically significant differences were observed in either COVID-19 morbidity or mortality by treatment type. In contrast, another study concluded that cancer patients who had received antineoplastic treatments including chemotherapy, radiotherapy, targeted therapy, and immunotherapy within 14 days before COVID-19 diagnosis were at higher risk of developing serious adverse events (63). In response to this, two meta-analyses suggested that chemotherapy increases the risk of death in cancer patients with COVID-19, while immunotherapy, targeted therapy, surgery, and radiotherapy showed no significant differences in safety (98, 99).

In addition, immune checkpoint inhibitors (ICIs) have gained increasing attention because of their potential impact on the disease course of COVID-19 in cancer immunotherapy, where they either act directly on immune checkpoints and are able to enhance the immune response or relieve immunosuppression (100, 101) (**Table 1**). Studies have confirmed that the use of ICIs in patients with lung cancer does not affect symptom severity during COVID-19 (103). Meanwhile, Hanna et al. (107) found that prolonged doses of ICIs had similar efficacy and toxicity compared with standard doses, which provided a reference for extended dosing regimens to reduce the risk of viral exposure triggered by visits in patients with lung cancer. Indeed, accumulating evidence suggests (108, 109) that ICIs may exert antiviral effects by enhancing T cell (CD4+ and CD8+) levels and activity, improving clinical outcomes in critically ill patients. It remains to be noted that during COVID-19, ICIs may trigger an excessive release of cytokines, contributing to a systemic inflammatory disease, and we recommend close attention to therapeutic reflexes of patients.

**Table 1:** Summary of the impact of immune checkpoint inhibitors on lung cancer combined with Corona Virus Disease 2019 (COVID-19).

Publication time	Patient number	Disease features	Treatment	Conclusion	Ref.
2020 Jun 12	54	76% NSCLC, 15% Small-cell lung cancer	63% ICIs, 37% Chemotherapy+ICIs	Immunotherapy did not worsen outcomes for patients with cancer with COVID-19 in our analysis	(102)
2020 May 12	69	93% NSCLC, 7% Small-cell lung cancer	Anti-PD1/PDL-1	PD-1 blockade exposure was not associated with increased risk of severity of COVID-19.	(103)
2021 Jun 26	1	Metastatic lung cancer	Anti-PD1+chemotherapy+pemetrexed	Discharge in an improved overall condition, and no sign of recurring pneumonitis.	(104)
2021 Feb 16	159	34% NSCLC	86.2% anti-PD1/PDL-1, 12.6% anti-CTLA4+anti-PD1, 1.2% anti-CTLA4	The mortality rate is higher in SARS-CoV-2-positive patients treated with ICIs.	(105)
2022 Feb 19	228	41.7% Lung cancer	25%ICIs+chemotherapy, 75% ICIs	The use of ICIs before SARS-CoV-2 infection does not affect COVID-19 severity or survival outcomes.	(106)

Ref., References; Jun, January; NSCLC, Non-small cell lung cancer; ICIs, immune checkpoint inhibitors; PD-1/PD-L1, the programmed death-1/programmed death ligand-1; Feb, February; CTLA-4, Cytotoxic T lymphocyte-associated antigen-4.

## 6 Conclusions

Lung cancer is one of the most threatening malignancies to population health, and its relevance began to be recognised after COVID-19 caused a public health emergency that swept the globe. Analysis data showed that patients with lung cancer had a poor prognosis, higher rates of death (HR = 2.00 [95% CI 1.52, 2.63], p < 0.01) and severe infection (HR = 1.47 [95%CI 1.06, 2.03], p = 0.02) compared to patients without cancer (110). As discussed here, the onset, progression, and prognosis of the two diseases are not isolated and may, in some cases, affect each other and are causal. For example, lung cancer is a risk factor for COVID-19 infection and adverse outcomes, and the COVID-19 pandemic may worsen the condition in lung cancer patients (111–113). Among them, inflammation is an important link; it has a bifacial nature and can not only promote immune responses but also lead to immunosuppression. Some scholars have suggested (114) that although the pathological manifestations of acute inflammation and chronic inflammation are different, the driving mechanism of the two is relevant. As such, starting with acute inflammatory diseases may improve the diagnosis and treatment of chronic inflammation; conversely, therapies developed for chronic diseases may be beneficial for acute inflammation. Given the complex relationship between lung cancer and COVID-19, we think it is necessary to gain further insight into the roles and effects of acute and chronic inflammation-related cytokines, chemokines, and inflammatory mediators to provide more scientific treatment options and a basis for patients.

## Author contributions

YA, Conceptualization, Supervision, Writing – original draft, Writing – review & editing. HW, Conceptualization, Supervision, Writing – original draft, Writing – review & editing. QZ, Conceptualization, Supervision, Writing – original draft, Writing – review & editing. SL, Writing, original draft, Writing – review & editing. JL Writing, original draft, Writing – review & editing. JH, Validation, Supervision, Writing – original draft, Writing – review & editing. JT, Conceptualization, Supervision, Writing – original draft, Writing – review & editing. XM, Conceptualization, Supervision, Writing – original draft, Writing – review & editing. All authors contributed to the article and approved the submitted version.
